# Analysis of Molecular Mechanism of Erxian Decoction in Treating Osteoporosis Based on Formula Optimization Model

**DOI:** 10.1155/2021/6641838

**Published:** 2021-06-18

**Authors:** Lang Yang, Liuyi Fan, Kexin Wang, Yupeng Chen, Lan Liang, Xuemei Qin, Aiping Lu, Peng Cao, Bin Yu, Daogang Guan, Junxiang Peng

**Affiliations:** ^1^Department of Neurosurgery, Nanfang Hospital, Southern Medical University, Guangzhou, China; ^2^Department of Orthopaedics and Traumatology, Nanfang Hospital, Southern Medical University, Guangzhou, China; ^3^Modern Research Center for Traditional Chinese Medicine, Shanxi University, Taiyuan, China; ^4^Institute of Integrated Bioinformedicine and Translational Science, Hong Kong Baptist University, Hong Kong, China; ^5^Department of Biochemistry and Molecular Biology, School of Basic Medical Sciences, Southern Medical University, Guangzhou, China; ^6^Guangdong Provincial Key Laboratory of Single Cell Technology and Application, Guangzhou, China; ^7^Department of Computer Science, Hong Kong Baptist University, Hong Kong SAR, China; ^8^Department of Surgery, Chinese People's Liberation Army 96608 Military Hospital, China

## Abstract

Osteoporosis (OP) is a highly prevalent orthopedic condition in postmenopausal women and the elderly. Currently, OP treatments mainly include bisphosphonates, receptor activator of nuclear factor kappa-B ligand (RANKL) antibody therapy, selective estrogen receptor modulators, teriparatide (PTH1-34), and menopausal hormone therapy. However, increasing evidence has indicated these treatments may exert serious side effects. In recent years, Traditional Chinese Medicine (TCM) has become popular for treating orthopedic disorders. Erxian Decoction (EXD) is widely used for the clinical treatment of OP, but its underlying molecular mechanisms are unclear thanks to its multiple components and multiple target features. In this research, we designed a network pharmacology method, which used a novel node importance calculation model to identify critical response networks (CRNs) and effective proteins. Based on these proteins, a target coverage contribution (TCC) model was designed to infer a core active component group (CACG). This approach decoded the mechanisms underpinning EXD's role in OP therapy. Our data indicated that the drug response network mediated by the CACG effectively retained information of the component-target (C-T) network of pathogenic genes. Functional pathway enrichment analysis showed that EXD exerted therapeutic effects toward OP by targeting PI3K-Akt signaling (hsa04151), calcium signaling (hsa04020), apoptosis (hsa04210), estrogen signaling (hsa04915), and osteoclast differentiation (hsa04380) via JNK, AKT, and ERK. Our method furnishes a feasible methodological strategy for formula optimization and mechanism analysis and also supplies a reference scheme for the secondary development of the TCM formula.

## 1. Introduction

Osteoporosis (OP) is a metabolic bone disorder characterized by bone-mass reduction, bone microstructural degeneration, increased bone fragility, decreased bone strength, and increased fracture risk [1]. Approximately 33% of elderly women and 20% of elderly men suffer from osteoporotic fractures. OP not only leads to the loss of health but also leads to societal social burdens and economic losses. Currently, several pharmacological products are available for OP therapy; hormone replacement therapy (HRT) and bisphosphonates are mainly used for bone loss conditions, including OP. However, prolonged HRT use may significantly elevate the risks for endometrial and breast cancer, coronary heart disease, and other cardiovascular diseases. Similarly, bisphosphonates may induce necrosis of long bones and the jaw [2]. These serious complications severely limit clinic use of these therapies. Therefore, it is necessary to find new alternative treatments with less side effects in the treatment of osteoporosis. Traditional Chinese Medicine (TCM) approaches have been successfully used to treat OP, including Erxian Decoction [3–5], Zuogui Pill [6–8], and Liuwei Dihuang Pill [9–11]. A recesnt meta-analysis of 644 patients with OP showed that EXD was therapeutically beneficial [12] and its different components elicited curative effects toward OP [5, 13, 14].

EXD consists of 6 herbs: *Curculigo orchioides* Gaertn. (“Xian Mao”, XM, 15 g), *Epimedium brevicornu* Maxim. (“Yin-Yang-Huo”, YYH, 15 g), *Angelica sinensis* (Oliv.) Diels (“Dang Gui”, DG, 9 g), *Morinda officinalis* F.C. How (“Ba-Ji-Tian”, BJT, 9 g), *Phellodendron chinense* C.K. Schneid (“Huang Bai”, HB, 9 g), and *Anemarrhena asphodeloides* Bunge (“Zhi Mu”, ZM, 9 g). Of these herbal components, BJT is rich in saccharides, iridoids, organic acids, and other components, which are believed to enhance immunity and reproductive functions, improve OP, and mediate depression and oxidation [15]. In a recent study, BJT and its extract strongly promoted MC3T3-E1 (mouse osteoblasts) proliferation [16]. Monotropein and inulin are the main BJT extraction components and their anti-OP effects have been verified in several studies [16, 17]. Similarly, XM also exerts antioxidant properties, anticancer potential, and may be used as an anti-OP herb. The XM components, curculigoside, curculigoside A, thalassoin glucoside, and thalassoin gentiobioside may also promote osteoblast proliferation [18]. YYH comes from the dried leaves of *Epimedium* and is widely used in TCM formulae to treat bone diseases [19]. Current pharmacological research has shown that YYH improves OP by downregulating PGE2 and ADR*β*-2R expression [20]. Also, DG has been combined with other herbs to improve postmenopausal OP symptoms in women, e.g., in EXD and Danggui Buxue Tang [21–23]. Additionally, ZM is derived from the *Anemarrhena asphodeloides Bge* rhizome and has antitumor, antioxidant, antimicrobial, antiviral, anti-inflammatory, and anti-OP effects [24]. Pharmacological studies confirmed that ZM limited reductions in trabecular thickness, increased the trabecular separation of proximal tibia metaphysis, and promoted bone formation in ovariectomized rats [25].

EXD is a multicomponent and multitargeting biological formula, which could form a complex drug response network in the treatment process. Critically, some of the relationships in this network have key roles in treatments, whereas others have auxiliary roles. Therefore, determining these core active components is critical to our understanding of the EXD molecular mechanisms implicated in OP therapy.

Network pharmacology is a newly emerging discipline integrating systems biology, polypharmacology, computational biology, network analysis, and other disciplines. Combined, the approach provides greater insights on the molecular mechanisms of drug actions from an overall system perspective, reflective of complex diseases and associated treatments. In recent years, the approach has been used to decode TCM mechanisms [26], e.g., Wang et al. used a collaborative network model to analyze Zhizhu pill mechanisms for dyspepsia therapy [27]. Tao et al. exploited network pharmacology to investigate active components and potential targets of *radix curcumae* formulae to treat cardiovascular disease [28].

The core active component group (CACG) and its mediated critical response network (CRN) refer to pharmacologically active components and targets in Chinese herb formula which are closely associated with positive therapeutic disease responses. Determining key CACG for specific disease treatments is difficult thanks to high chemical composition complexity and a limited understanding of complex multitargeting TCM mechanisms. By selecting key CACG for TCM, we can eliminate nonactive components from Chinese herb treatments and focus on key TCM treatment mechanisms.

In this research, a network pharmacology module was implemented to determine CACG and elucidate EXD therapeutic mechanisms for OP. We constructed a weighted gene regulatory network for OP which was used for CRN construction and analysis. After this, we gathered EXD components from different databases and potential active components were selected by using published ADME-related models. Targets of these active components were predicted using three online prediction tools. The active components and their target were used to generate the component-target (C-T) network. The weighted gene regulatory network and C-T network were used to construct a component-target-pathogenic (C-T-P) network. The node importance calculation method was designed to assess critical response networks and determine effective proteins. A target coverage contribution (TCC) model was designed to screen CACG based on selected effective proteins from the CRN. Finally, chosen CACG were used to characterize EXD molecular mechanisms implicated in OP therapy.

## 2. Materials and Methods

### 2.1. Construction of a Weighted Gene Regulatory Network for OP

Potential OP pathogenetic genes were extracted from the GeneCards database (https://www.genecards.org/). Protein-protein interaction (PPI) datasets were downloaded from CMGRN [29], PTHGRN [30], BioGRID (https://thebiogrid.org), and STRING [31] databases. All PPI datasets were merged into a comprehensive PPI dataset by removing duplicates. Potential OP pathogenetic genes were mapped to the comprehensive PPI network, and the weight of each pathogenetic gene was assigned a “relevance score” which was stored in the GeneCards database. The network was visualized using Cytoscape (version 3.5.1).

### 2.2. EXD Component Collection

EXD components were collected from four natural product sources: TCMSP [32], TCM integrated database [33], TCM database@Taiwan [34], and YaTCM [27]. For EXD components, structures (e.g., mol2 and SDF) were transformed to unified SMILE formats using the Open Babel toolkit (version 2.4.1). Then, component characteristics were retrieved from TCMSP, including molecular weight (MW), oral bioavailability (OB), Caco-2 permeability (Caco-2), and DL (drug-likeness).

### 2.3. Selecting Potentially Active EXD Components Based on ADME Models

ADME characteristics, including OB, MW, Caco-2, and DL, were used to screen bioactive molecules. Based on previous reports [35–37], we selected OB > 30%, MW < 500 Da [38], Caco‐2 > 0.4 [39], and DL > 0.14 [28] as screening criteria for active compounds. These criteria will also be incorporated with ADME screening in future research.

### 2.4. Predicting Active Component Targets

To identify EXD active component targets, chemical structures were assembled into canonical SMILES. The online tools, Similarity Ensemble Approach (SEA) [28], HitPick [40], and Swiss Target Prediction [41] were used to identify targets.

### 2.5. Defining CRN and Evaluating Effective Proteins

We designed a method to characterize the importance of nodes; therefore, we defined Net_ctp_ = {*N*, *E*}, where *N* = nodes representing components, targets, and pathogenic genes and *E* = edges representing interactions in the C-T-P network:

(1) $$\begin{array}{c} W_{i}=\sqrt{\frac{1}{\sum_{k-1}^{n-1}d\left(i,k\right)}}\times \frac{\sum_{j}^{n}\sum_{k}^{n}\left(t_{jk}\left(i\right)/t_{jk}\right)}{n\left(n-1\right)/2},\\ W_{\text{avg}}=\text{avg}\left\{W_{1},W_{2},W_{3,}\cdots W_{n}\right\},\\ \text{CRN}=\overset{n}{\underset{i=1}{\cup }}W_{\left({\text{Net}}_{\text{ctp}}\right)i}>W_{\text{avg}}, \end{array}$$

where *W*_*i*_ is the importance of node *i* in the network; *t*_*jk*_ is the path numbers between nodes *j* and *k*. *t*_*jk*_(*i*) is the number of paths from node *j* to node *k*, and through node *i*. *d*(*i*, *k*) is the shortest path from node *i* to node *k*, and *n* is the total number of nodes in the network. CRN is the critical response network. Nodes in the CRN were assigned as effective proteins.

### 2.6. Developing a TCC Model to Select CACG

We optimized effective components and identified CACG, which were used to illustrate potential EXD molecular mechanisms for OP therapy. Active components associated with effective proteins were extracted as *κ* = {*κ*_1_, *κ*_2_, *κ*_3_ ⋯ ⋯*κ*_*m*_}. The target number of each active components was defined as *ξ* = {*ξ*_1_, *ξ*_2_, *ξ*_3_ ⋯ ⋯*ξ*_*m*_},; then, the coverage of the target number for each active components was defined as *v* = {*v*_1_, *v*_2_, *v*_3_ ⋯ ⋯*v*_*m*_}, and the TCC model based on dynamic-0-1 knapsack principle is described in Algorithm 1.

### 2.7. Gene Ontology and Pathway Analysis

To analyze target functions, the clusterProfiler package, R software [42] was used to perform Gene Ontology (GO) and Kyoto Encyclopedia of Genes and Genomes (KEGG) [43] pathway enrichment analysis. For data processing, *p* values < 0.05 were set as the cut-off. The ggplot2 package was used to create graphs in R statistical programming language (version 3.4.2). Data analysis was annotated by Pathview [44] in the R Bioconductor package (https://www.bioconductor.org/).

## 3. Results

**Figure 1:**
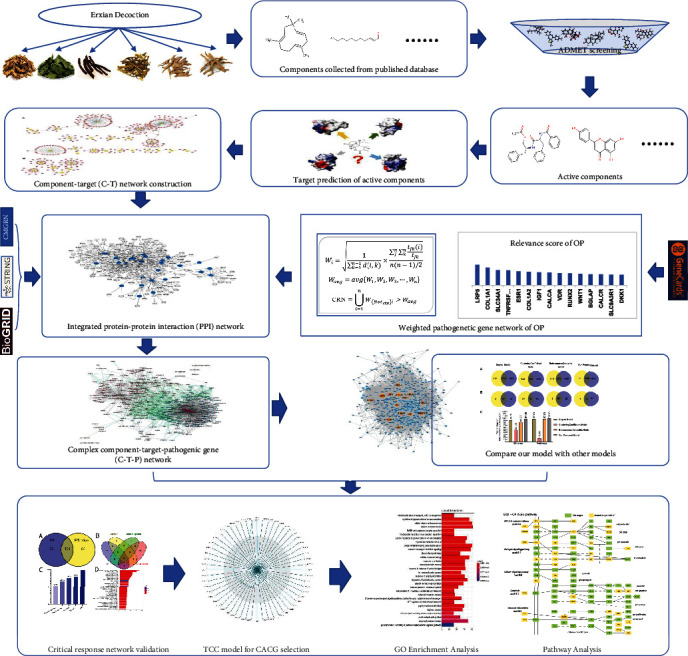
The network pharmacology approach. The following workflow was used in our proposed approach: (1) Known EXD components were collected from databases and active components derived by ADME screening. (2) All target genes of active ingredients were identified by using the prediction tools (SEA, HitPick, and Swiss Target Prediction), and a component-target (C-T) network was constructed. (3) OP pathogenic genes were identified from GeneCards and genes selected with ^“^relevance scores^”^ > 5 to construct a pathogenic gene network. (4) Through PPI network analysis of the C-T and pathogenic gene networks, a component-target-pathogenic gene (C-T-P) network was derived. (5) A node importance calculation method was then designed to capture the critical response network (CRN) and effective proteins. Then, the Kyoto Encyclopedia of Genes and Genomes pathway and Gene Ontology (GO) analyses were used to validate the CRN and effective proteins. (6) The TCC model was designed to select the core active component group (CACG) from the critical network. (7) Underlying EXD mechanisms for OP were deduced based on CACG.

We developed a network pharmacology-based strategy to capture CACG and decode underlying EXD molecular mechanisms for OP therapy (Figure 1). Specifically, a weighted OP pathogenetic gene network was constructed based on “relevance scores.” Also, all EXD components were extracted from online databases. Then, putative active components were screened from all EXD components based on previous ADME methods. Active component targets were predicted using published tools, and active components and targets were also used to generate a C-T network. The weighted pathogenetic gene network and the C-T network were mapped to our comprehensive PPI network to generate a complex C-T-P network. Also, a node importance calculation method was designed to identify the CRN from the C-T-P network. After this, a TCC model was designed to select CACG, and finally, CACG were used to predict EXD molecular mechanisms for OP therapy.

### 3.1. Construction of Weighted Gene Regulatory OP Networks

**Figure 2:**
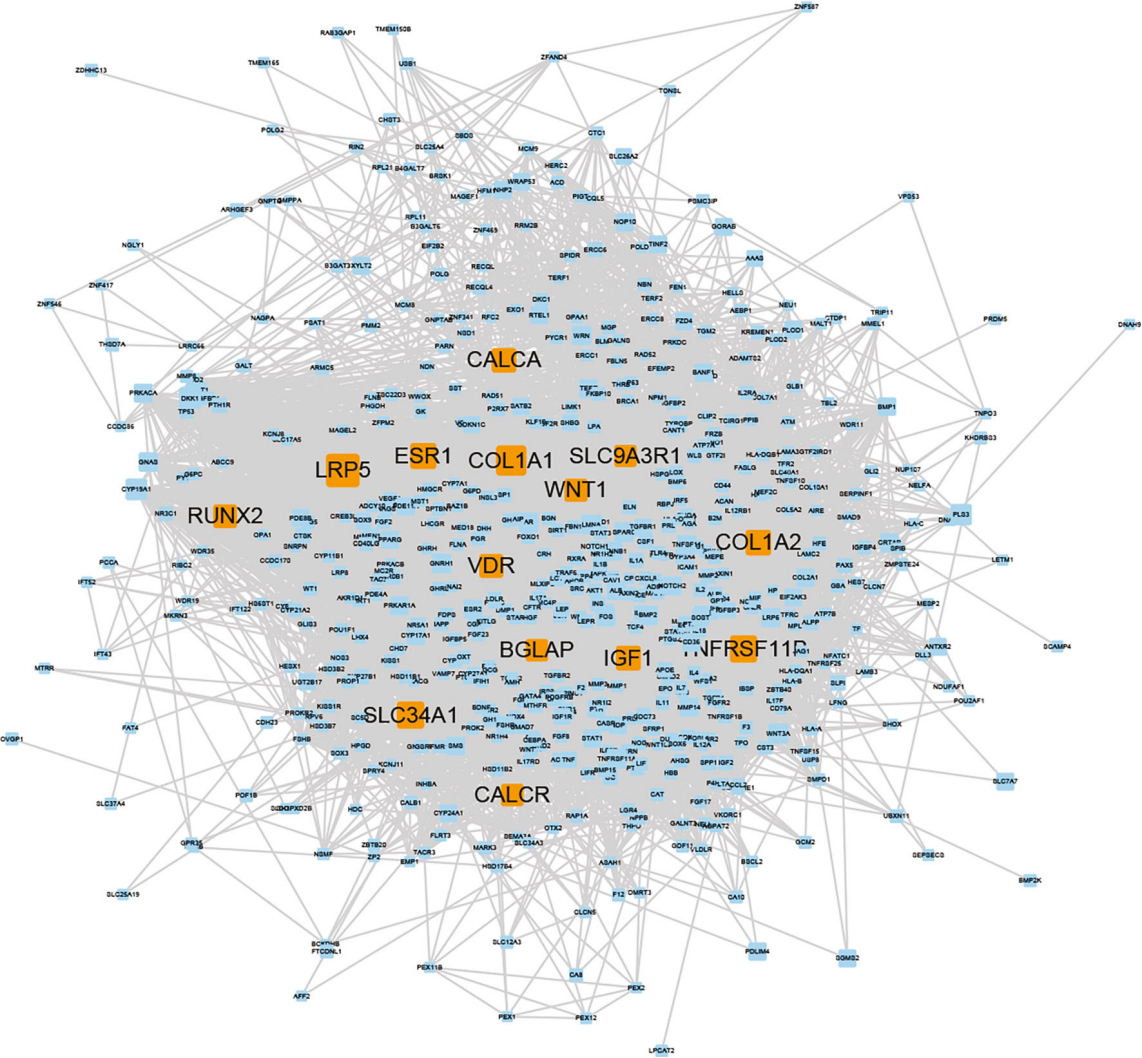
Weighted OP pathogenetic gene network. Node size represents “relevance scores,” and the orange nodes represent ^“^relevance scores^”^ > 40.

Weighted gene regulatory network construction and analytical approaches are key to understanding OP pathogenesis and therapeutic intervention strategies. To construct a comprehensive weighted OP gene network, PPI datasets from CMGRN, PTHGRN, BioGRID, and STRING were used to establish the PPI network. Approximately 660 genes with ^“^relevance scores^”^ > 5 were selected (Supplementary Table 1) and mapped to the PPI network to generate a weighted OP gene regulatory network. After removing isolated nodes, the network contained 592 nodes and 11320 edges (Figure 2). Genes with ^“^relevance scores^”^ > 40 were considered pathogenic genes with high correlation to OP and included LRP5 [45, 46], COL1A1 [47, 48], SLC34A1, TNFRSF11B [49], ESR1 [50, 51], COL1A2 [52, 53], IGF1 [54], CALCA, VDR [55, 56], RUNX2 [57, 58], WNT1 [59, 60], BGLAP [61], CALCR [61, 62], and SLC9A3R1 [63]. For example, LRP5 had the highest OP “relevance scores,” and its pathogenic risk for OP has been identified in different races [64]. The ESR1 gene polymorphism leads to decreased bone mineral density in postmenopausal women [51] and predicts OP occurrence in women with Crohn's disease [65]. The single nucleotide polymorphisms, 245T > G and 1181G > C in TNFRSF11B, were associated with lumbar spine bone mineral density in OP postmenopausal women and possibly altered susceptibility to OP [49]. These data suggested our weighted gene regulatory network and weighted genes indicated OP pathogenesis genes and provided a reliable reference for CRN construction.

### 3.2. Herb Components in EXD

Using a systematic investigation of six herb components from EXD, 600 components were identified (Table 1). More information is provided (Supplementary Table 2).

### 3.3. The Selection of Potential Active Components

While TCM formulae contain many components, only a few elicit acceptable pharmacodynamic and pharmacokinetic characteristics. In this research, four ADME-related models, comprising MW, OB, Caco-2, and DL, were used to screen and identify potential active components.

Chemical analyses are commonly used to study effective herb components and mechanisms. From the literature, we identified high concentration components in EXD (references included in Table 2). These components typically elicit potent biological activities; therefore, we combined components after ADME screening with these high-concentration components to generate 183 active components (Table 1). More information is shown (Supplementary Table 3).

### 3.4. Shared and Specific Herb Components in EXD

**Figure 3:**
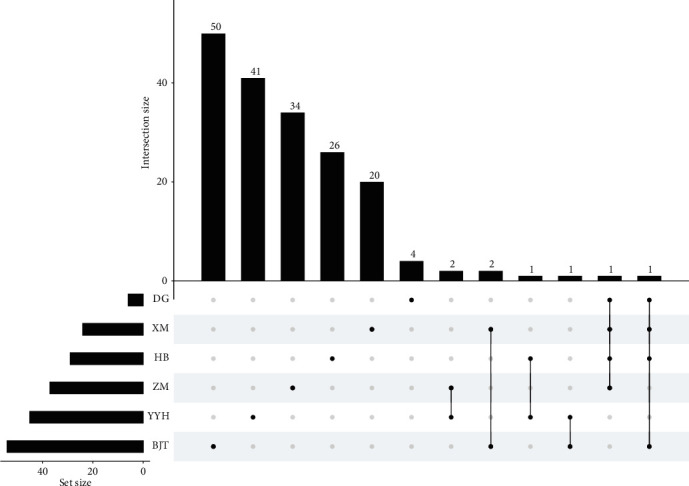
The frequency distributions of common and specific components in six herbs of EXD. UpSetR was used to map and plot points representing herbs, and corresponding histograms represented active component frequencies. Unconnected black dots referred to specific herb components. Linked dots represented shared active components.

As indicated (Supplementary Table 2), eight active components were shared by two or more EXD herbs (Figure 3), e.g., *β*-sitosterol (EXD8) is a component of five herbs including HB, BJT, DG, YYH, and XM. Stigmasterol (EXD13) is shared by ZM, HB, DG, and XM. Magnoflorine (EXD24) is shared by HB and YYH. Apart from shared components, most herbs possessed definitive components, e.g., diosgenin (EXD17), berberine (EXD 30), kaempferol (EXD12), and curculigenin A (EXD88) were specific components of ZM, HB, XM, and YYH, respectively. These data revealed that EXD potentially exerted therapeutic effects toward OP by orchestrating shared and specific components.

### 3.5. Predicting Active Component Targets

To identify EXD therapeutic mechanisms for OP therapy, 183 active components were used to predict targets using SEA, HitPick, and Swiss Target. After this, 1027 targets were predicted. Then, the 183 active and 1027 targets were used to construct a C-T network (Supplementary Table 4). Some components were related to multiple targets, resulting in 8328 component-target associations between the 183 active components and 1027 targets. The average number of targets per component was 33.16. Of these, lignoceric acid (EXD20, degree = 182) had the highest number of targets, followed by rubiadin-1-methyl ether (EXD181, degree = 158), xuelianlactone (EXD130, degree = 131), arachic acid (EXD3, degree = 126), oleanolic acid (EXD6, degree = 126), luteolin (EXD1, degree = 125), (+)-syringaresinol (EXD10, degree = 124), apigenin (EXD2, degree = 117), and *β*-sitosterol (EXD8, degree = 114). Most components were related to calcium homeostasis and apoptosis-related OP pathways [72–77]. These data demonstrated the important roles of these components in OP therapy and further confirmed that EXD functioned in a multicomponent manner to treat OP.

From the C-T network, the average degree of targets for different components was 13.79. The top 20 ranking targets with larger weights included ESR1, ESR2, CYP1B1, and SHBG. The majority of these targets were associated with OP pathogenesis and potentially indicated EXD therapeutic mechanisms for OP. These data suggested EXD functioned in a multitargeted manner as an OP therapy.

### 3.6. Effective Protein Selection and Validation Using the CRN

We employed the weighted gene regulatory network and active component target network to construct a C-T-P network. This comprised 1344 nodes and 30790 edges. The importance of nodes in networks is a critical topological evaluation property of networks. Here, we designed a novel node importance calculation method to assess the important score of each node in the C-T-P network. For each node, if the important score of a node was more than the average important score of all nodes in the network, the node was believed to play a critical role in network structure and was treated as a critical node [78]. Following this rule, the important score of each node in the C-T-P network was calculated and compared with the average important score of all nodes in the network. Passed nodes and their edges in the C-T-P network were retained and defined as a CRN. This CRN contained 1344 nodes and 15395 edges, and one node represented one effective protein; therefore, 530 effective proteins were identified from the CRN.

**Figure 4:**
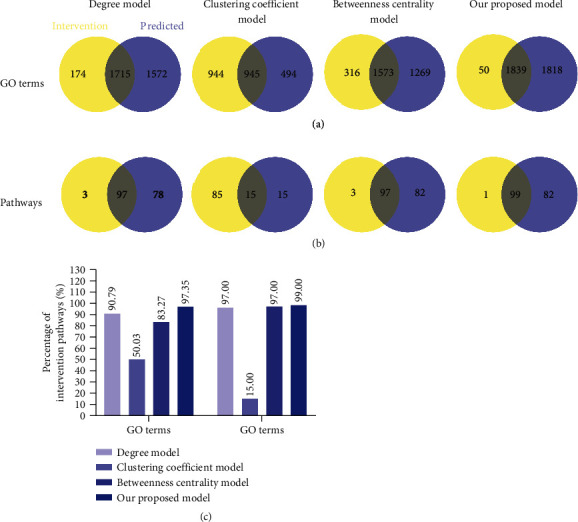
Compare our proposed model with other widely used models. (a) Venn diagrams display the number of overlapped GO terms of four models with main intervention GO terms. (b) Venn diagrams display the number of overlapped pathways of four models with intervention pathways, respectively. (c) Comparison of our model with other models on the intervention pathways and GO terms.

To verify the reliability and accuracy of our proposed node importance calculation method for constructing CRN, we conduct enrichment pathway analysis of EXD target genes and pathogenic genes and processed the intersection part of pathways as intervention pathways. Similarly, we select the overlapped part of GO enrichment of EXD targets and pathogenic genes and defined it as the intervention GO terms. Based on the calculation results of our method, the enriched pathway and GO term of 530 effective proteins account for 99% and 97.35% of the intervention pathways and GO terms, respectively (Figure 4 and Table S5). This result shows that our model can retain the targets with intervention potential to the greatest extent at the functional coverage level.

In order to further prove the reliability and accuracy of our model, we compare our proposed node importance calculation method with other commonly used node importance calculation methods, including degree, betweenness centrality, and clustering coefficient. We use our model and these models to obtain respective effective proteins and use these effective proteins to perform pathway and GO enrichment analysis, respectively, and then check the percentages of effective protein-enriched pathways and GO terms in intervention pathways and intervention GO terms, respectively. The higher the percentage, the higher the reliability and accuracy of the model. Results show that the percentage of effective protein-enriched pathways found in our model in intervention pathway and intervention GO term is significantly higher than that in the degree model, betweenness centrality model, and clustering coefficient model. These results show that compared with other node importance models, our model has higher accuracy and better functional coverage.

**Figure 5:**
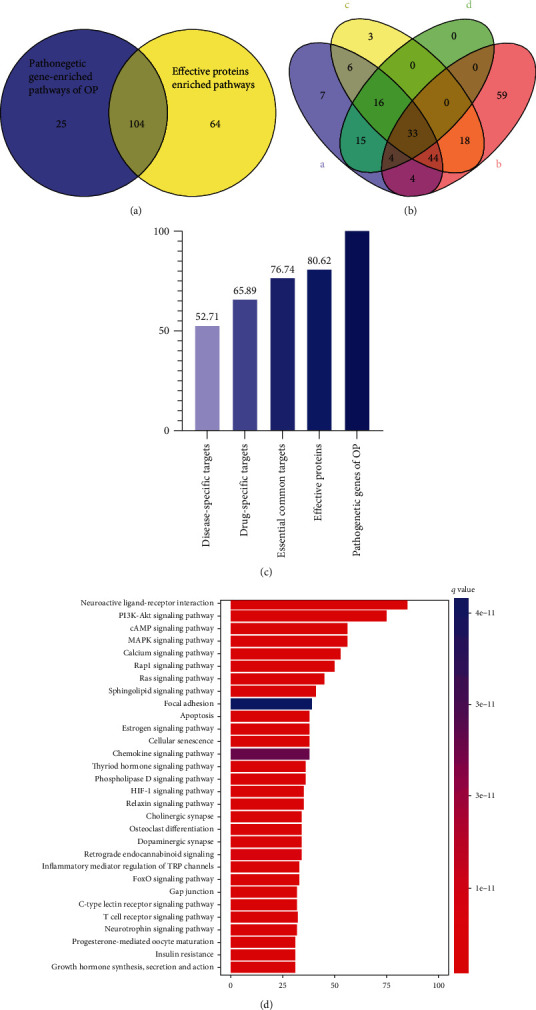
Critical response network validation. (a) Venn diagram shows enriched pathways of effective proteins and pathogenetic genes of OP; (b) Venn diagram displays enrichment pathways of essential component-specific targets, disease-specific targets, essential common targets, and pathogenetic genes of OP; (c) the coverage proportion of component-specific targets, disease-specific targets, common targets, and effective protein-enriched pathways compared with pathogenetic gene-enriched pathways of OP; (d) barplot for effective protein-enriched pathways.

Three effective protein categories were present in CRNs. The first comprised direct interactions between component targets and pathogenic genes and was defined as essential common targets. The second category covered disease-specific target interactions, and the third category encompassed component-specific target interactions. To assess whether effective proteins in CRN could be substituted by disease-specific targets, component-specific targets or essential common targets were identified for optimization. We conducted pathway analysis on essential common targets, disease-specific targets, and component-specific targets. We showed that the coverage proportion of enriched pathways of targets in three categories when compared with enrichment pathways of pathogenic genes was 52.71%, 65.89%, and 76.74%, respectively (Figures 5(b) and 5(c)). These were all lower than the coverage proportion of effective protein-enriched pathways, which account for 80.62% enrichment pathways of pathogenic genes. These data verified the model accuracy and reliability in constructing a CRN and showed that effective proteins from the CRN had important roles in OP pathogenesis.

Based on pathway enrichment analyses, effective proteins were involved in PI3K-Akt signaling (hsa04151), calcium signaling (hsa04020), apoptosis (hsa04210), estrogen signaling (hsa04915), and osteoclast differentiation (hsa04380) (Figure 5(d)). The pathway enrichment analysis and document evidence revealed that most effective proteins are associated with cell proliferation and apoptosis, which are confirmed related to the osteoblasts and osteoclasts of OP and that may be the underlying mechanism of EXD in treating OP.

### 3.7. CACG Selection and Validation

**Figure 6:**
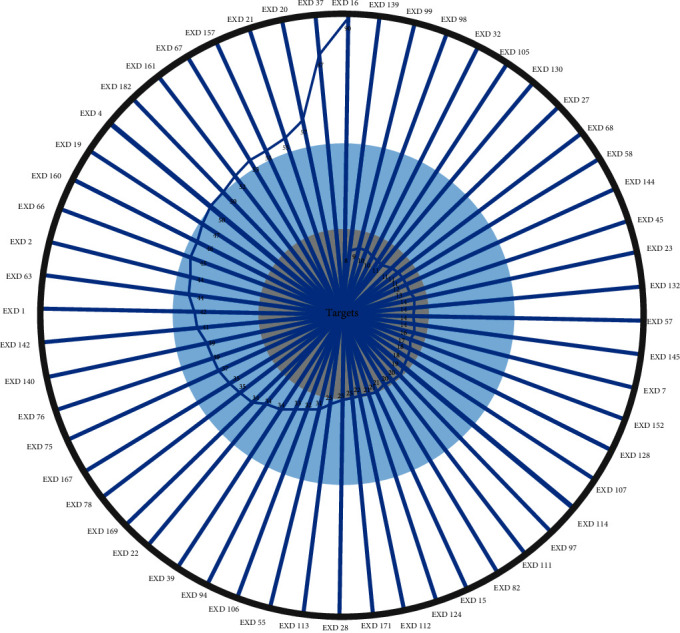
Accumulative TCC scores of EXD active components. TCC scores were calculated according to the cumulative contribution rate of targets.

The TCC model was established to optimize the CRN and get the CACG which were used to determine EXD molecular mechanisms for OP treatment. Based on the contribution accumulation calculation results, the top five components included EXD16 ((Z)-3-(4-hydroxy-3-methoxy-phenyl)-N-[2-(4-hydroxyphenyl) ethyl]acrylamide), EXD37 (asperglaucide), EXD20 (lignoceric acid), EXD21 (oleic acid), and EXD157 (1-hydroxy-3-methoxy-9,10-anthraquinone), and the targets of the five components accounted for 47.92% of effective proteins. Furthermore, targets of 37 components and 56 components contributed to 90.38% and 100% of effective proteins; therefore, we selected these latter 56 components as CACG (Figure 6 and Table 2). This higher effective protein coverage indicated CACG may have critical therapeutic roles and generate combination effects for OP treatments.

**Figure 7:**
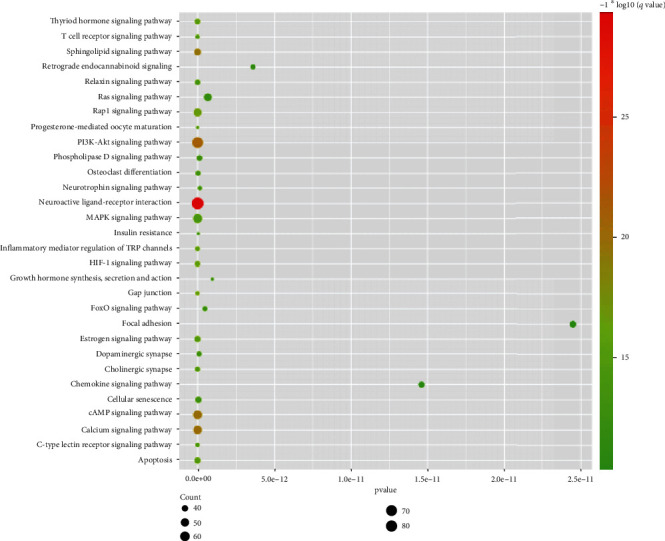
Pathway enrichment analysis of CACG targets for EXD. The list of top 30 enriched pathways.

To analyze EXD effects toward OP treatment at the functional level, pathway analyses were conducted (Figure 7) using CACG targets and OP pathogenic genes. CACG target-enriched pathways numbered 186 (*p* < 0.05), and pathogenic gene-enriched pathways numbered 129 (*p* < 0.05). CACG target-enriched pathways covered 77.52% of pathogenic gene-enriched pathways. These CACG-mediated targets were enriched in neuroactive ligand-receptor interactions (hsa04080), PI3K-Akt signaling (hsa04151), cAMP signaling (hsa04024), MAPK signaling (hsa04010), estrogen signaling (hsa04915), osteoclast differentiation (hsa04380), etc. These results indicated that combining the CRN with the TCC model to optimize herbal formulae was reliable, and predicted CACG potentially triggered therapeutic roles by identifying cell proliferation-related osteoblast and osteoclast pathways.

### 3.8. GO Enrichment Analysis of CACG Targets

**Figure 8:**
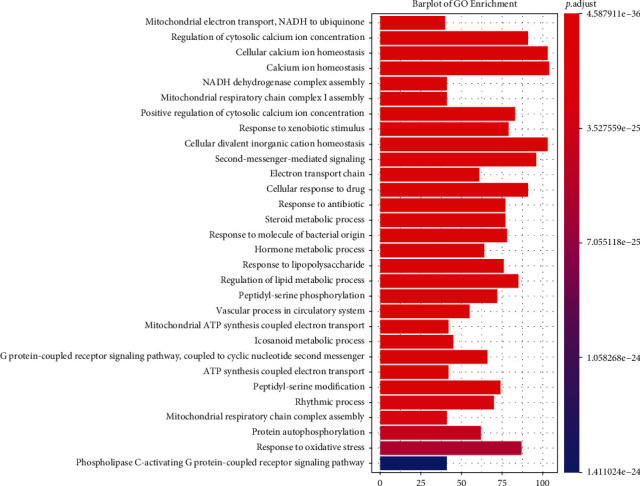
GO enrichment processing of CACG targets. The list of top 30 enriched GO terms.

To decode EXD combination effects, all CACG targets were enriched by GO analyses (Figure 8). This approach indicated that targets regulated by CACG of EXD were enriched in calcium ion regulation processes, e.g., cytosolic calcium ion concentration pathways (GO:0051480, ADRA1B, BCL2, C3AR1, HTR2A, etc.), cellular calcium ion homeostasis (GO:0006874, CCR2, DRD4, GRIN2D, NMUR2, etc.), calcium ion homeostasis (GO:0055074, CCR5, EDNRA, LPAR1, PIK3CB, etc.), and positive regulation of cytosolic calcium ion concentrations (GO:0007204, CXCR2, FYN, LPAR3, S1PR3, etc.). These GO data verified that EXD was effective for OP via calcium regulation and apoptosis mechanisms.

### 3.9. Pathway Enrichment Analysis of CACG Targets

**Figure 9:**
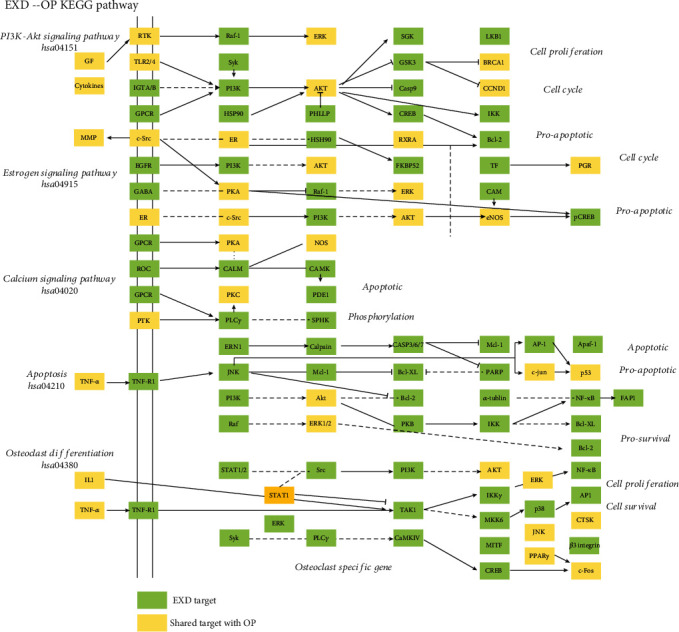
Distribution of CACG targets in EXD on the compressed OP pathway. The green nodes refer to unique CACG targets. Yellow nodes represent common CACG targets and OP pathogenic genes.

For this analysis, we identified 100 pathways shared by CACG and pathogenic genes, including PI3K-Akt signaling (hsa04151), estrogen signaling (hsa04915), and osteoclast differentiation (hsa04380). While calcium signaling (hsa04020) and apoptosis (hsa04210) were important processes in OP pathogenesis based on the literature. Increasing evidence has indicated these pathways are related to OP pathogenesis or are therapy targets for OP. To identify EXD mechanisms for OP therapy from a system perspective, we generated an integrated signaling pathway using five molecular pathways (Figure 9).

To define EXD target pathway positions, we considered the first three columns as upstream and the rest as downstream positions of the integrated pathway. PI3K-Akt signaling (hsa04151) was identified as one of the top pathways implicated in OP treatment with EXD. EXD regulated 24 targets upstream of PI3K-Akt signaling (hsa04151), e.g., RTK, TLR2/4, IGTA/B, and GCPR, and 55 targets in downstream pathways, e.g., PI3K, AKT, and ERK. EXD may be putatively activated downstream of the PI3K and AKT proteins via upstream TLR2/4, resulting in a downstream GSK3, CCND1, and BCL-2 cascade, which are closely related to bone cell proliferation and apoptosis. Most EXD targets regulating estrogen signaling (hsa04915) were downstream of the pathway, e.g., PKA, AKT, and RAF-1. Also, we observed (Figure 9) that EXD activated nuclear-activated steroid signaling to exert important roles for OP treatment. Therefore, EXD elicits key therapeutic functions for OP treatment via ER-AKT/ERK cascade regulation to synergistically affect cell cycle and proapoptosis processes.

Calcium signaling (hsa04020) and apoptosis (hsa04210) are vital pathways in OP treatments mediated by EXD. Those targets regulated by EXD are downstream of the integrated pathway. For example, EXD affected upstream GPCR, ROC, and CALM and also activated downstream CAMK to affect OP-mediated apoptosis in the calcium signaling pathway (hsa04020). Additionally, 26 EXD targets, including BCL-2, CASP3/6/7, and P53, were located downstream of the apoptosis pathway (hsa04210). EXD also activated downstream JNK, AKT, and ERK via upstream TNF-*α*, and TNFR affect a series of prosurvival and apoptosis process of bone-related cell. 18 of 35 genes in osteoclast differentiation pathways are enriched in the apoptosis pathway (including AKT1/2/3, RELA, MAPK1/3/8/9/10, FOS, JUN, NFKB1, PIK3CD, PIK3CA, PIK3CB, IKBKG, TNFRSF1A, and CTSK), and 14/35 genes in osteoclast differentiation pathways are also enriched in the PI3K/AKT pathway (including AKT1/2/3, RELA, MAPK1/3, NFKB1, PIK3CD, PIK3CA, PIK3CB, IKBKG, CREB1, SYK, and ITGB3). Recently, a multiscale embedded gene coexpression network analysis (MEGENA) suggested that osteoclast differentiation may contribute to postmenopausal OP pathogenesis [79]. Combined, these data suggested apoptosis and PI3K/AKT pathways could elicit key effects on osteoclast differentiation and implied that EXD regulated osteoclast processes mainly through these pathways.

## 4. Discussion

The fundamental mechanism underlying OP involves imbalanced bone homeostasis related to osteoblast-osteoclast coupling. Currently, OP therapeutics mainly include bone formation promoters, bone resorption inhibitors, and bone mineral agents, e.g., strontium ranelate, teriparatide acetate, bisphosphonate, and calcium agents. However, some of these drugs have low specificity, high costs, and considerable side effects. TCM strategies have been used for OP treatment because they are less toxic and have limited side effects. Many EXD ingredients exert significant therapeutic effects toward OP, to effectively promote bone formation, inhibit bone absorption, and increase bone density. TCM is advantageous for treating complex diseases, as it is comprised of many components and focuses on many targets. TCM constitutes a highly complex regulatory network and involves synergistic effects, antagonistic effects, and also certain toxic side effects. Thus, optimizing these relationships, determining key synergistic components, and removing toxicity and side effects are viable starting points and main drivers of TCM component optimization.

Network pharmacology analyses, using a systems biology approach to improve our understanding of drug behavior on cell and organ at the molecular level, accelerate the identification of drug targets and importantly help discover new biomarkers. Using this approach, scientists can systematically predict and explain drug effects, optimize drug design, determine factors affecting drug effectiveness and safety, and thus design multitarget drugs or drug combinations. However, few studies have used network pharmacology to optimize TCM formulae to identify functional components. Currently, scientists mine gene, protein, metabolite, and microorganism data from different experimental approaches to share information in assorted public databases. This approach is highly beneficial for analyzing pathogenic and therapeutic targets of disease. Such network pharmacology strategies and high-throughput data facilitate the exploration of TCM therapeutic mechanisms for complex disease treatment.

In this report, we designed a network pharmacology strategy to optimize CACG in EXD for OP therapy and to identify hidden molecular mechanisms. The model included a new node importance calculation method and a dynamic programming algorithm. The CRN and effective proteins were derived based on the new node importance calculation method, with the CACG derived using a dynamic programming algorithm according to effective proteins in CRN. Finally, underlying mechanisms of EXD toward OP were determined based on CACG. When compared with other network pharmacology strategies, our strategy had three main advantages.

The first was that we used a new node importance algorithm to extract CRN and effective proteins to treat OP with EXD. The important feature of this algorithm was that the importance of nodes in the network was related to the bridging ability and leadership of nodes. The bridging ability of nodes referred to the number of times a node acted as the shortest bridge between two other nodes. The higher the number of times a node acted as a “bridge,” the greater its bridging abilities. We divided the number of times a node spanned the shortest bridge by the number of paths. When the bridging ability of one node was high, this meant that many or even all of the shortest paths between other nodes must have transited through it. If the node disappeared, communication between other nodes would have become difficult and possibly disconnected. Leadership referred to the radiation and control ability of a node in the whole network. Specifically, in a network, leadership may be interpreted as the probability that a node influences or radiates (to) other nodes in the network. When compared with the average leadership of the network, leadership nodes could easily become the adjustment center of other nodes, and nodes with very high leadership were more likely to organize functional units or modules to influence other nodes. If the leadership was high, the distance between these nodes and every other node in the neighborhood was very short when compared with their diameter, which was easy to form radiation and influence on other nodes. If node leadership was low, this meant the node was peripheral or isolated.

To our surprise, the enrichment pathways and GO terms of effective proteins determined by our method accounted for 99% and 97.35% of intervention pathways and intervention GO terms, respectively. To test whether effective proteins in a CRN could be substituted by disease-specific targets, component-specific targets, or essential common targets for further optimization, the pathway enrichment analysis of effective proteins, disease-specific targets, component-specific targets, and essential common targets showed that the ratio of effective protein-enriched pathways to pathogenic gene-enriched pathways was as high as 80.62%, which is 27. 91%, 14.73%, and 5.88% higher than that of other three datasets, respectively. These data proved that our method retained as many potential therapeutic targets as possible and also confirmed method reliability and accuracy. In addition, when comparing our method with other calculation methods such as degree, betweenness centrality, and clustering coefficient models, we observed that the enrichment pathways of effective proteins captured by our method accounted for 99% of intervention pathways, which was higher than comparator models, at 2%, 2%, and 84%, respectively. The enriched GO terms of effector proteins of our method accounted for 97.35% of intervention GO terms, which was 6.56%, 14.08%, and 47.32% higher than comparator models, respectively. These data validated the reliability and accuracy of our method.

Secondly, we used the TCC model to screen CACG based on effective proteins, which was an effective approach. Our analyses showed that the number of CACG target-enriched pathways was 186 (*p* < 0.05), and the pathogenic gene-enriched pathways numbered 129 (*p* < 0.05). CACG target-enriched pathways covered 77.52% of pathogenic gene-enriched pathways.

The third advantage was that our data were supported by the literature. The results of shared and specific components of herbs in EXD provided a basis for CACG optimization, e.g., *β*-sitosterol (EXD8) is a common component of five herbs including HB, BJT, DG, YYH, and XM and strongly regulates apoptosis processes [80, 81] which are important during OP treatments. Stigmasterol (EXD13), shared by ZM, HB, DG, and XM, regulated cell proliferation [82] and apoptosis [83]. It has also been revealed that stigmasterol could attenuate cell calcium concentration. Magnoflorine (EXD24), shared by HB and YYH, not only protected osteoblasts against oxidative damage from ERK5/Nrf2 signaling [84] but also attenuated osteoclastogenesis and bone resorption. In a rat model, metabonomic analyses showed that rubiadin-1-methyl (EXD181) ether ameliorated OP by regulating arachidonic acid metabolism [85]. Furthermore, Wu et al. reported that EXD181 inhibited osteoclast TRAP activity and promoted osteoblast proliferation [86]. Diosgenin (EXD17) is a specific component of ZM and reduced bone mass loss in ovariectomized rats by lowering RANKL/OPG ratios [87] and improving the level of estrogenic hormone of estradiol [88]. Berberine (EXD 30) is a specific component of HB and was shown to exert antioxidant effects through the RANK/RANKL/OPG pathway to reduce OP development in rats [89]. Kaempferol (EXD12) has a powerful role in bone protection via regulation of the estrogen receptor, bone morphogenetic protein-2 (BMP-2), nuclear factor-kappa B (NF-*κ*B), mitogen-activated protein kinase (MAPK), and mammalian target of rapamycin (mTOR) signaling pathways [90]. Curculigenin A (EXD88) is a major component of YYH and promoted osteoblast proliferation by increasing alkaline phosphatase activity in osteoblasts [91]. Therefore, these components may be considered therapeutic components for OP.

According to our C-T network analyses, most of these components were reportedly related to OP-mediated calcium homeostasis and apoptosis pathways, e.g., *β*-sitosterol effectively inhibited osteoclast differentiation [77] and promoted a time- and dose-dependent increase of calcium uptake in activated neutrophils [74]. Moreover, *β*-sitosterol induced apoptosis in several cell lines [72, 73, 75, 76]. Goto et al. reported that apigenin completely inhibited multinucleated osteoclast formation [92]. Apigenin also stimulated increased cytosolic calcium ion concentrations in bovine artery endothelial cells [93]. Zhao et al. also observed that apigenin inhibited proliferation and induced apoptosis in human melanoma cells [94]. Luteolin prevented bone loss in postmenopausal OP by reducing the differentiation ability of osteoclasts [95]. Also, luteolin inhibited L-type calcium currents in ventricular myocytes under hypoxia conditions and ameliorated calcium ion overload in rat cardiomyocytes [96].

By analyzing EXD targets, we hypothesize that EXD acts synergistically to mediate OP via calcium regulation and apoptosis, e.g., several studies have shown that ESR1 variants are related to postmenopausal OP [97–99], and ESR1 gene polymorphisms may be predictive of OP in female patients with Crohn's disease [65]. ESR2 variants have been shown to increase fracture risks in postmenopausal women [100] and could affect renal calcium oxalate crystal deposition [101]. In addition, according to a multilocus analysis of estrogen-related genes, an interactive role was determined for ESR1, ESR2, and NRIP1 genes in OP pathogenesis [102]. Splicing defects in CYP1B1 also appeared to alter calcium channel functions [103], whereas a CYP1B1 polymorphism was speculated to be a possible genetic risk factor for OP in American women [104]. High SHBG levels are an independent risk factor for OP in men [105], especially in those with prostate cancer [106].

To explore potential EXD mechanisms for OP treatment, CACG targets were used for GO enrichment analyses. We showed that targets regulated by EXD were enriched in calcium ion regulation processes. Also, some other genes involved in apoptosis pathways included BCL2, HSP90B1, and PIK3CB, with higher associations with OP [107–109]. Adult bone quality maintenance is not only controlled by osteoclast and osteoblast production changes but also by apoptosis regulation to alter cellular life spans. Osteoblast and osteoclast dysregulation is also an important factor leading to OP development [110]. Our GO investigations indicated that EXD ameliorated OP via calcium regulation and apoptosis processes.

OP is an age-related disease associated with estrogen in postmenopausal women. Its clinical manifestations are bone mass reduction and bone structure destruction, which are related to calcium ion regulation. As important mechanical force sensors, G-protein-coupled receptors, via calcium ion and cAMP signaling, appear to influence osteocyte mechanobiology behaviors. PI3K-Akt signaling, estrogen signaling, osteoclast differentiation, calcium signaling, and apoptosis pathways were selected to construct comprehensive pathways to illustrate EXD mechanisms in OP. Increasing evidence has indicated these pathways are related to OP pathogenesis or therapy targets. For example, PI3K-Akt signaling (hsa04151) mainly regulates protein synthesis, cell proliferation, and apoptosis. Besides regulating cell proliferation and apoptosis, calcium signaling (hsa04020) also regulates metabolism. Apoptosis is a genetically programmed process which eliminates damaged or redundant cells via caspase (aspartate-specific cysteine proteases) activation. The balance between pro- and antiapoptotic signals ultimately determines whether cells undergo apoptosis, survival, or proliferation. Estrogen is expressed via “nuclear-initiated steroid signaling” and “membrane-initiated steroid signaling” regulation and includes a plethora of physical processes in mammals including reproduction, cardiovascular protection, bone integrity, cellular homeostasis, and behavior. Several herbs have demonstrated anti-OP roles by regulating osteoblast proliferation and osteoclast differentiation (hsa04380) [16, 111–113].

In this study, the network pharmacology model proposed by us has good advantages in using algorithms to remove noise and retain CRN; our network pharmacology model exploited algorithms to remove noise and retain CRN, using dynamic programming to select key component groups and verifying model reliability by using the reliable documentary evidence. Thus, our model is a powerful tool for investigating TCM compatibility and mechanisms in disease. Our study had some limitations, e.g., we should have considered more major active components during our EXD research. However, going forward, we will corroborate our active ingredient data for OP treatments using *in vivo* and/or *in vitro* studies.

## Figures and Tables

**Algorithm 1:**
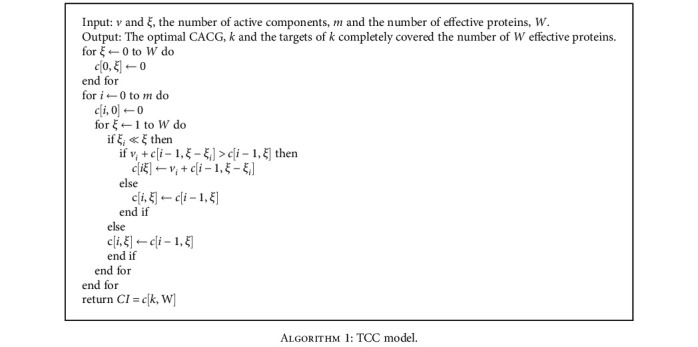
TCC model.

**Table 1:** EXD herbal components identified and collected from databases.

Herbs	Overall	Active components
*Anemarrhena asphodeloides* Bunge (ZM)	79	37
Curculigo orchioides Gaertn (XM)	77	24
*Phellodendron chinense* C.K. Schneid (HB)	58	29
*Morinda officinalis* F.C. How (BJT)	174	54
*Epimedium brevicornu* maxim (YYH)	129	45
Angelica sinensis (Oliv.) Diels (DG)	125	6
Total	600	183

**Table 2:** High-concentration EXD components identified from the literature.

Formula	Method	Component	Concentration	Ref.
ZM	HPLC	Mangiferin	32.30 mg/g	[66]
		Timosaponin B-II	17.70 mg/g	

BJT	RP-HPLC	2-Hydroxy-3-hydroxymethylanthraquinone	94.36 *μ*g/g	[67]
		2-Hydroxy-1-methoxy anthranquinone	164.43 *μ*g/g	
		Rubiadin-1-methyl ether	80.26 *μ*g/g	
		Rubiadin	21.53 *μ*g/g	

XM	HPLC	5-Hydroxymethylfurfural	0.028~0.151 mg/g	[68]
		2-Hydroxy-5-(2-hydroxyethyl) phenyl-*β*-D-glucopyranoside	0.031~0.338 mg/g	
		Anacardoside	0.207~3.172 mg/g	
		Orcinol glucoside	1.795~6.239 mg/g	
		Orcinol-1-O-*β*-D-apiofuranosyl-(1 → 6)-*β*-D-glucopyranoside	0.150~2.088 mg/g	
		2,6-Dimethoxybenzoic acid	0.504~1.790 mg/g	
		Curculigoside	0.919~2.187 mg/g	
		Curculigine A	0.066~0.262 mg/g	

YYH	HPLC	Total flavone	80.7 ± 2.11 mg/g	[69]
		Epimedium lan	11.1 ± 0.25 mg/g	

DG	HPLC	Ferulic acid	0.20~0.24 mg/g	[70]

HB	HPLC	Berberine	6.63 mg/g	[71]
		Phellodendrine	87.7 mg/g	

## Data Availability

In order to construct a comprehensive weight gene network of OP, the potential pathogenetic genes of OP were extracted from GeneCards databases (https://www.genecards.org/). The PPI datasets were downloaded from CMGRN, PTHGRN, BioGRID (https://thebiogrid.org), and STRING databases. All components of EXD were collected from four published natural product data sources: TCMSP database, Traditional Chinese Medicine integrated database, Traditional Chinese Medicine database@Taiwan, and YaTCM.

## Abbreviations

CACG: Core active component group
DL: Drug-likeness
EXD: Erxian Decoction
GO: Gene Ontology
KEGG: Kyoto Encyclopedia of Genes and Genomes
OP: Osteoporosis
OB: Oral bioavailability
PPI: Protein-protein interactions
TCC: Target coverage contribution
TCM: Traditional Chinese Medicine.

## Supplementary Materials

Supplementary Materials Table 1: the collected pathogenic genes of OP, relevance score more than 5. Table 2: the collected components of EXD. Table 3: the active components of EXD. Table 4: the interactions of active components and their targets. Table 5: effective proteins and CACG.

## References

[1] Black D. M., Rosen C. J. (2016). Postmenopausal osteoporosis. *The New England Journal of Medicine*.

[2] Khosla S., Hofbauer L. C. (2017). Osteoporosis treatment: recent developments and ongoing challenges. *The lancet Diabetes & Endocrinology*.

[3] Wang N., Xin H., Xu P., Yu Z., Shou D. (2019). Erxian decoction attenuates TNF-*α* induced osteoblast apoptosis by modulating the Akt/Nrf2/HO-1 signaling pathway. *Frontiers in Pharmacology*.

[4] Wang N., Xu P., Wang X. (2019). Integrated pathological cell fishing and network pharmacology approach to investigate main active components of Er-Xian decotion for treating osteoporosis. *Journal of Ethnopharmacology*.

[5] Wong K. C., Lee K. S., Luk H. K. (2014). Er-xian decoction exerts estrogen-like osteoprotective effects in vivo and in vitro. *The American Journal of Chinese Medicine*.

[6] Chen G., Zhang Z., Liu Y. (2019). Efficacy and safety of Zuogui pill in treating osteoporosis: study protocol of a systematic review. *Medicine*.

[7] Wenxiong L., Kuaiqiang Z., Zhu L. (2018). Effect of Zuogui pill and Yougui pill on osteoporosis: a randomized controlled trial. *Journal of Traditional Chinese Medicine*.

[8] Yin H., Wang S., Zhang Y., Wu M., Wang J., Ma Y. (2018). Zuogui pill improves the dexamethasone-induced osteoporosis progression in zebrafish larvae. *Biomedicine & Pharmacotherapy*.

[9] Li M., Wang W., Wang P., Yang K., Sun H., Wang X. (2010). The pharmacological effects of morroniside and loganin isolated from Liuweidihuang Wan, on MC3T3-E1 cells. *Molecules*.

[10] Ge J. R., Xie L. H., Chen J. (2018). Liuwei Dihuang pill () treats postmenopausal osteoporosis with shen (kidney) yin deficiency via Janus kinase/signal transducer and activator of transcription signal pathway by up-regulating cardiotrophin-like cytokine factor 1 expression. *Chinese Journal of Integrative Medicine*.

[11] Xu F., Gao F. (2018). Liuwei Dihuang pill cures postmenopausal osteoporosis with kidney-yin deficiency: potential therapeutic targets identified based on gene expression profiling. *Medicine*.

[12] Li J.-Y., Jia Y.-S., Chai L.-M. (2017). Effects of Chinese herbal formula Erxian decoction for treating osteoporosis: a systematic review. *Clinical Interventions in Aging*.

[13] Nian H., Qin L. P., Zhang Q. Y., Zheng H. C., Yu Y., Huang B. K. (2006). Antiosteoporotic activity of Er-Xian Decoction, a traditional Chinese herbal formula, in ovariectomized rats. *Journal of Ethnopharmacology*.

[14] Qin L., Han T., Zhang Q. (2008). Antiosteoporotic chemical constituents from Er-Xian decoction, a traditional Chinese herbal formula. *Journal of Ethnopharmacology*.

[15] Feng Y. (2019). Development of modern pharmacological research on the antidepressant effects of euphordolitids. *Journal of Liaoning University of Traditional Chinese Medicine*.

[16] Zhang Z., Zhang Q., Yang H. (2016). Monotropein isolated from the roots of Morinda officinalis increases osteoblastic bone formation and prevents bone loss in ovariectomized mice. *Fitoterapia*.

[17] Jiang K., Huang D., Zhang D. (2018). Investigation of inulins from the roots of *Morinda officinalis* for potential therapeutic application as anti-osteoporosis agent. *International Journal of Biological Macromolecules*.

[18] Zhang N. D., Jiang Y. P., Xue L. M., Han T., Zhang Q. Y., Xin H. L. (2016). Phenolic glycosides in Curculigo orchioides promotes osteoblastic bone formation and inhibits osteoclastic bone resorption. *School of Pharmacy, Second Military Medical University*.

[19] Wang L., Li Y., Guo Y. (2016). Herba Epimedii: an ancient Chinese herbal medicine in the prevention and treatment of osteoporosis. *Current Pharmaceutical Design*.

[20] Dong C., Shi J., Chen X. (2019). Beneficial effects of Herba Epimedii on ovariectomized rats by down-regulating PGE2 and ADR*β*-2R expression. *Minerva Medica*.

[21] Gong A. G. W., Duan R., Wang H. Y., Dong T. T. X., Tsim K. W. K. (2018). Calycosin orchestrates osteogenesis of Danggui Buxue Tang in cultured osteoblasts: evaluating the mechanism of action by omics and chemical knock-out methodologies. *Frontiers in Pharmacology*.

[22] Xie Q. F., Xie J. H., Dong T. T. (2012). Effect of a derived herbal recipe from an ancient Chinese formula, Danggui Buxue Tang, on ovariectomized rats. *Journal of Ethnopharmacology*.

[23] Zhou L. P., Wong K. Y., Yeung H. T. (2018). Bone protective effects of Danggui Buxue Tang alone and in combination with tamoxifen or raloxifene in vivo and in vitro. *Frontiers in Pharmacology*.

[24] Wang Y., Dan Y., Yang D. (2014). The genus Anemarrhena Bunge: a review on ethnopharmacology, phytochemistry and pharmacology. *Journal of Ethnopharmacology*.

[25] Nian H., Qin L. P., Chen W. S., Zhang Q. Y., Zheng H. C., Wang Y. (2006). Protective effect of steroidal saponins from rhizome of Anemarrhena asphodeloides on ovariectomy-induced bone loss in rats1. *Acta Pharmacologica Sinica*.

[26] Hopkins A. L. (2008). Network pharmacology: the next paradigm in drug discovery. *Nature Chemical Biology*.

[27] Wang C., Ren Q., Chen X. T. (2018). System pharmacology-based strategy to decode the synergistic mechanism of Zhi-zhu Wan for functional dyspepsia. *Frontiers in Pharmacology*.

[28] Tao W., Xu X., Wang X. (2013). Network pharmacology-based prediction of the active ingredients and potential targets of Chinese herbal Radix Curcumae formula for application to cardiovascular disease. *Journal of Ethnopharmacology*.

[29] Guan D., Shao J., Deng Y. (2014). CMGRN: a web server for constructing multilevel gene regulatory networks using ChIP-seq and gene expression data. *Bioinformatics*.

[30] Guan D., Shao J., Zhao Z. (2014). PTHGRN: unraveling post-translational hierarchical gene regulatory networks using PPI, ChIP-seq and gene expression data. *Nucleic Acids Research*.

[31] Szklarczyk D., Gable A. L., Lyon D. (2019). STRING v11: protein-protein association networks with increased coverage, supporting functional discovery in genome-wide experimental datasets. *Nucleic Acids Research*.

[32] Ru J., Li P., Wang J. (2014). TCMSP: a database of systems pharmacology for drug discovery from herbal medicines. *Journal of Cheminformatics*.

[33] Xue R., Fang Z., Zhang M., Yi Z., Wen C., Shi T. (2013). TCMID: Traditional Chinese Medicine integrative database for herb molecular mechanism analysis. *Nucleic Acids Research*.

[34] Chen C. Y. (2011). TCM Database@Taiwan: the world’s largest traditional Chinese medicine database for drug screening in silico. *PLoS One*.

[35] Wang K. X., Gao Y., Gong W. X. (2020). A novel strategy for decoding and validating the combination principles of Huanglian Jiedu decoction from multi-scale perspective. *Frontiers in Pharmacology*.

[36] Wang K. X., Gao Y., Lu C. (2020). Uncovering the complexity mechanism of different formulas treatment for rheumatoid arthritis based on a novel Pharmacology Model. *Frontiers in Pharmacology*.

[37] Gao Y., Wang K. X., Wang P. (2020). A Novel Network Pharmacology Strategy to Decode Mechanism of Lang Chuang Wan in Treating Systemic Lupus Erythematosus. *Frontiers in Pharmacology*.

[38] Lipinski C. A., Lombardo F., Dominy B. W., Feeney P. J. (2001). Experimental and computational approaches to estimate solubility and permeability in drug discovery and development settings. *Advanced Drug Delivery Reviews*.

[39] Shultz M. D. (2019). Two Decades under the Influence of the Rule of Five and the Changing Properties of Approved Oral Drugs. *Journal of Medicinal Chemistry*.

[40] Liu X., Vogt I., Haque T., Campillos M. (2013). HitPick: a web server for hit identification and target prediction of chemical screenings. *Bioinformatics*.

[41] Gfeller D., Grosdidier A., Wirth M., Daina A., Michielin O., Zoete V. (2014). SwissTargetPrediction: a web server for target prediction of bioactive small molecules. *Nucleic Acids Research*.

[42] Yu G., Wang L. G., Han Y., He Q. Y. (2012). clusterProfiler: an R package for comparing biological themes among gene clusters. *OMICS: A Journal of Integrative Biology*.

[43] Draghici S., Khatri P., Tarca A. L. (2007). A systems biology approach for pathway level analysis. *Genome Research*.

[44] Luo W., Pant G., Bhavnasi Y. K., Blanchard S. G., Brouwer C. (2017). Pathview Web: user friendly pathway visualization and data integration. *Nucleic Acids Research*.

[45] Ciubean A. D., Ungur R. A., Irsay L. (2019). Polymorphisms of FDPS, LRP5, SOST and VKORC1 genes and their relation with osteoporosis in postmenopausal Romanian women. *PLoS One*.

[46] Luther J., Yorgan T. A., Rolvien T. (2018). Wnt1 is an Lrp5-independent bone-anabolic Wnt ligand. *Science Translational Medicine*.

[47] Majchrzycki M., Bartkowiak-Wieczorek J., Wolski H. (2015). Polymorphisms of collagen 1A1 (COL1A1) gene and their relation to bone mineral density in postmenopausal women. *Ginekologia Polska*.

[48] Wu J., Yu M., Zhou Y. (2017). Association of collagen type I alpha 1 +1245G/T polymorphism and osteoporosis risk in post-menopausal women: a meta-analysis. *International Journal of Rheumatic Diseases*.

[49] Mencej-Bedrač S., Preželj J., Marc J. (2011). TNFRSF11B gene polymorphisms 1181G>C and 245T>G as well as haplotype CT influence bone mineral density in postmenopausal women. *Maturitas*.

[50] Luo L., Xia W., Nie M. (2014). Association of ESR1 and C6orf97 gene polymorphism with osteoporosis in postmenopausal women. *Molecular Biology Reports*.

[51] Mondockova V., Adamkovicova M., Lukacova M. (2018). The estrogen receptor 1 gene affects bone mineral density and osteoporosis treatment efficiency in Slovak postmenopausal women. *BMC Medical Genetics*.

[52] Jiang Z. S., Hao Z. H. (2016). An insertion/deletion polymorphism within the 3′-untranslated region of COL1A2 confers susceptibility to osteoporosis. *Molecular Medicine Reports*.

[53] Majchrzycki M., Bartkowiak-Wieczorek J., Bogacz A. (2017). The importance of polymorphic variants of collagen 1A2 gene (COL1A2) in the development of osteopenia and osteoporosis in postmenopausal women. *Ginekologia Polska*.

[54] Chen X. W., Li Y. H., Zhang M. J. (2019). Lactoferrin ameliorates aging-suppressed osteogenesis via IGF1 signaling. *Journal of Molecular Endocrinology*.

[55] Nakamichi Y., Udagawa N., Horibe K. (2017). VDR in Osteoblast-Lineage Cells Primarily Mediates Vitamin D Treatment-Induced Increase in Bone Mass by Suppressing Bone Resorption. *Journal of Bone and Mineral Research: the Official Journal of the American Society for Bone and Mineral Research*.

[56] Zhang L., Yin X., Wang J. (2018). Associations between VDR Gene Polymorphisms and Osteoporosis Risk and Bone Mineral Density in Postmenopausal Women: A systematic review and Meta-Analysis. *Scientific Reports*.

[57] Cheng F., Yang M. M., Yang R. H. (2019). MiRNA-365a-3p promotes the progression of osteoporosis by inhibiting osteogenic differentiation via targeting RUNX2. *European Review for Medical and Pharmacological Sciences*.

[58] Feng J., Wang J. X., Li C. H. (2019). LncRNA GAS5 overexpression alleviates the development of osteoporosis through promoting osteogenic differentiation of MSCs via targeting microRNA-498 to regulate RUNX2. *European Review for Medical and Pharmacological Sciences*.

[59] Laine C. M., Joeng K. S., Campeau P. M. (2013). WNT1 mutations in early-onset osteoporosis and osteogenesis imperfecta. *The New England Journal of Medicine*.

[60] Mäkitie R. E., Hackl M., Niinimäki R., Kakko S., Grillari J., Mäkitie O. (2018). Altered MicroRNA Profile in Osteoporosis Caused by Impaired WNT Signaling. *The Journal of Clinical Endocrinology and Metabolism*.

[61] Tural S., Kara N., Alayli G., Tomak L. (2013). Association between osteoporosis and polymorphisms of the bone Gla protein, estrogen receptor 1, collagen 1-A1 and calcitonin receptor genes in Turkish postmenopausal women. *Gene*.

[62] Lee H. J., Kim S. Y., Kim G. S. (2010). Fracture, bone mineral density, and the effects of calcitonin receptor gene in postmenopausal Koreans. *Osteoporosis International*.

[63] Dudakovic A., Evans J. M., Li Y. (2013). Histone deacetylase inhibition promotes osteoblast maturation by altering the histone H4 epigenome and reduces Akt phosphorylation. *The Journal of Biological Chemistry*.

[64] Xu G. Y., Qiu Y., Mao H. J. (2014). Common polymorphism in the LRP5 gene may increase the risk of bone fracture and osteoporosis. *BioMed Research International*.

[65] Krela-Kaźmierczak I., Skrzypczak-Zielińska M., Kaczmarek-Ryś M. (2019). ESR1 Gene Variants Are Predictive of Osteoporosis in Female Patients with Crohn's Disease. *Journal of Clinical Medicine*.

[66] Huang Q., Jia P., Xiao X., Fengqing X. U., Wu D. (2016). Comparison of the content of Mangiferin and Timosaponin B-II in the Fibril and Radix of *Anemarrhena Asphodeloides* by HPLC. *Clinical Journal of Traditional Chinese Medicine*.

[67] Bao L. L., Qin L. P., Zhang Q. Y., Bian J., Wu Y. (2010). Simultaneous Determination of 4 Kinds of Anthraquinones from Morinda officinalis by RP-HPLC. *China Pharmacy*.

[68] Wang Y., Liu L., Xu J. L., Guo Y. H., Zhang Q. Y. J. C. P. J. (2012). Simultaneous Determination of Phenolic Glycosides in Curculigins Rhizoma by HPLC. *Journal of Chinese Pharmaceutical Sciences*.

[69] Yu X., Jia H., Lei Z., Yang L., Jing S., Wang X. (2014). The content measure of total flavone and epimedium lan in Herba Epimedium get from different area of Gansu Province. *Journal of Gansu College of Traditional Chinese Medicine*.

[70] Yuan R. W., Zhao C. G., Xie L. M., Zheng M. W., Qin S. Z. (2014). Determination of Ferulic Acid Content in Angelica Sinensis by HPLC. *Guangzhou Chemical Industry*.

[71] Hong-Ying L. I., Xiang J. Q., Long L. (2013). Determination of Berberine and Phellodendrine in Cortex Phellodendri of Enshi by HPLC. *Journal of Hubei University for Nationalities*.

[72] Park C., Moon D. O., Ryu C. H. (2008). *β*-sitosterol sensitizes MDA-MB-231 cells to TRAIL-induced apoptosis. *Acta Pharmacologica Sinica*.

[73] Moon D. O., Lee K. J., Choi Y. H., Kim G. Y. (2007). *β*s-sitosterol-induced-apoptosis is mediated by the activation of ERK and the downregulation of Akt in MCA-102 murine fibrosarcoma cells. *International Immunopharmacology*.

[74] Liz R., Zanatta L., dos Reis G. O. (2013). Acute effect of *β*-sitosterol on calcium uptake mediates anti-inflammatory effect in murine activated neutrophils. *The Journal of Pharmacy and Pharmacology*.

[75] Sook S. H., Lee H. J., Kim J. H. (2014). Reactive oxygen species-mediated activation of AMP-activated protein kinase and c-Jun N-terminal kinase plays a critical role in beta-sitosterol-induced apoptosis in multiple myeloma U266 cells. *Phytotherapy Research : PTR*.

[76] Zhao Y., Chang S. K., Qu G., Li T., Cui H. (2009). *β*-sitosterol inhibits cell growth and induces apoptosis in SGC-7901 human stomach cancer cells. *Journal of Agricultural and Food Chemistry*.

[77] Zhang W., Xue K., Gao Y. (2019). Systems pharmacology dissection of action mechanisms of Dipsaci Radix for osteoporosis. *Life Sciences*.

[78] Liu H., Zeng L., Yang K., Zhang G. (2016). A Network Pharmacology Approach to Explore the Pharmacological Mechanism of Xiaoyao Powder on Anovulatory Infertility. *Evidence-based complementary and alternative medicine : eCAM*.

[79] Zhang L., Peng T. L., Wang L. (2020). Network-based Transcriptome-wide Expression Study for Postmenopausal Osteoporosis. *The Journal of Clinical Endocrinology & Metabolism*.

[80] Cao Z. Q., Wang X. X., Lu L. (2018). *β*-Sitosterol and Gemcitabine Exhibit Synergistic Anti-pancreatic Cancer Activity by Modulating Apoptosis and Inhibiting Epithelial-Mesenchymal Transition by Deactivating Akt/GSK-3*β* Signaling. *Frontiers in Pharmacology*.

[81] Rajavel T., Packiyaraj P., Suryanarayanan V., Singh S. K., Ruckmani K., Pandima Devi K. (2018). *β*-Sitosterol targets Trx/Trx1 reductase to induce apoptosis in A549 cells via ROS mediated mitochondrial dysregulation and p53 activation. *Scientific Reports*.

[82] Sriraman S., Ramanujam G. M., Ramasamy M., Dubey G. P. (2015). Identification of beta-sitosterol and stigmasterol in Bambusa bambos (L.) Voss leaf extract using HPLC and its estrogenic effect in vitro. *Journal of Pharmaceutical and Biomedical Analysis*.

[83] Li K., Yuan D., Yan R., Meng L., Zhang Y., Zhu K. (2018). Stigmasterol exhibits potent antitumor effects in human gastric cancer cells mediated via inhibition of cell migration, cell cycle arrest, mitochondrial mediated apoptosis and inhibition of JAK/STAT signalling pathway. *Journal of BUON : official journal of the Balkan Union of Oncology*.

[84] Xia G., Li X., Zhu X., Yin X., Ding H., Qiao Y. (2017). Mangiferin protects osteoblast against oxidative damage by modulation of ERK5/Nrf2 signaling. *Biochemical and Biophysical Research Communications*.

[85] Xia T., Dong X., Lin L. (2019). Metabolomics profiling provides valuable insights into the underlying mechanisms of Morinda officinalis on protecting glucocorticoid-induced osteoporosis. *Journal of Pharmaceutical and Biomedical Analysis*.

[86] Wu Y. B., Zheng C. J., Qin L. P. (2009). Antiosteoporotic activity of anthraquinones from Morinda officinalis on osteoblasts and osteoclasts. *Molecules*.

[87] Zhang Z., Song C., Fu X. (2014). High-dose diosgenin reduces bone loss in ovariectomized rats via attenuation of the RANKL/OPG ratio. *International Journal of Molecular Sciences*.

[88] Zhao S., Niu F., Xu C. Y. (2016). Diosgenin prevents bone loss on retinoic acid-induced osteoporosis in rats. *Irish Journal of Medical Science*.

[89] He X. F., Zhang L., Zhang C. H. (2017). Berberine alleviates oxidative stress in rats with osteoporosis through receptor activator of NF-kB/receptor activator of NF-kB ligand/osteoprotegerin (RANK/RANKL/OPG) pathway. *Bosnian Journal of Basic Medical Sciences*.

[90] Wong S. K., Chin K. Y., Ima-Nirwana S. (2019). The Osteoprotective Effects Of kaempferol: the evidence from in vivo and in vitro studies. *Drug Design, Development and Therapy*.

[91] Jiao L., Cao D. P., Qin L. P. (2009). Antiosteoporotic activity of phenolic compounds from Curculigo orchioides. *Phytomedicine: international journal of phytotherapy and phytopharmacology*.

[92] Goto T., Hagiwara K., Shirai N., Yoshida K., Hagiwara H. (2015). Apigenin inhibits osteoblastogenesis and osteoclastogenesis and prevents bone loss in ovariectomized mice. *Cytotechnology*.

[93] Chen C. C., Ke W. H., Ceng L. H., Hsieh C. W., Wung B. S. (2010). Calcium- and phosphatidylinositol 3-kinase/Akt-dependent activation of endothelial nitric oxide synthase by apigenin. *Life Sciences*.

[94] Zhao G., Han X., Cheng W. (2017). Apigenin inhibits proliferation and invasion, and induces apoptosis and cell cycle arrest in human melanoma cells. *Oncology Reports*.

[95] Kim T. H., Jung J. W., Ha B. G. (2011). The effects of luteolin on osteoclast differentiation, function in vitro and ovariectomy-induced bone loss. *The Journal of Nutritional Biochemistry*.

[96] Yan Q., Li Y., Yan J., Zhao Y., Liu Y., Liu S. (2019). Luteolin improves heart preservation through inhibiting hypoxia-dependent L-type calcium channels in cardiomyocytes. *Experimental and Therapeutic Medicine*.

[97] Greendale G. A., Wight R. G., Huang M. H. (2010). Menopause-associated symptoms and cognitive performance: results from the study of women’s health across the nation. *American Journal of Epidemiology*.

[98] Méndez J. P., Rojano-Mejía D., Coral-Vázquez R. M. (2013). Impact of genetic variants of IL-6, IL6R, LRP5, ESR1 and SP7 genes on bone mineral density in postmenopausal Mexican-Mestizo women with obesity. *Gene*.

[99] Shang D. P., Lian H. Y., Fu D. P., Wu J., Hou S. S., Lu J. M. (2016). Relationship between estrogen receptor 1 gene polymorphisms and postmenopausal osteoporosis of the spine in Chinese women. *Genetics and Molecular Research: GMR*.

[100] Rivadeneira F., van Meurs J. B., Kant J. (2006). Estrogen receptor *β* (ESR2) polymorphisms in interaction with estrogen receptor alpha (ESR1) and insulin-like growth factor I (IGF1) variants influence the risk of fracture in postmenopausal women. *Journal of Bone and Mineral Research: the Official Journal of the American Society for Bone and Mineral Research*.

[101] Zhu W., Zhao Z., Chou F. J. (2019). The protective roles of estrogen receptor *β* in renal calcium oxalate crystal formation via reducing the liver oxalate biosynthesis and renal oxidative stress-mediated cell injury. *Oxidative Medicine and Cellular Longevity*.

[102] Morón F. J., Mendoza N., Vázquez F. (2006). Multilocus analysis of estrogen-related genes in Spanish postmenopausal women suggests an interactive role of ESR1, ESR2 and NRIP1 genes in the pathogenesis of osteoporosis. *Bone*.

[103] Tang Z. Z., Yarotskyy V., Wei L. (2012). Muscle weakness in myotonic dystrophy associated with misregulated splicing and altered gating of Ca(V)1.1 calcium channel. *Human Molecular Genetics*.

[104] Napoli N., Rini G. B., Serber D. (2009). The Val432Leu polymorphism of the CYP1B1 gene is associated with differences in estrogen metabolism and bone density. *Bone*.

[105] Zha X. Y., Hu Y., Pang X. N., Zhu J. H., Chang G. L., Li L. (2014). Sex hormone-binding globulin (SHBG) as an independent determinant of bone mineral density (BMD) among Chinese middle-aged and elderly men. *Endocrine*.

[106] Varsavsky M., Reyes-García R., García-Martín A., González-Ramírez A. R., Avilés-Perez M. D., Muñoz-Torres M. (2013). SHBG levels are associated with bone loss and vertebral fractures in patients with prostate cancer. *Osteoporosis International: a journal established as result of cooperation between the European Foundation for Osteoporosis and the National Osteoporosis Foundation of the USA*.

[107] Zhao H., Wang L., Luo H., Li Q. Z., Zuo X. (2017). TNFAIP3 downregulation mediated by histone modification contributes to T-cell dysfunction in systemic lupus erythematosus. *Rheumatology*.

[108] Oates J. C., Levesque M. C., Hobbs M. R. (2003). Nitric oxide synthase 2 promoter polymorphisms and systemic lupus erythematosus in African-Americans. *The Journal of Rheumatology*.

[109] Marques C. P., Maor Y., de Andrade M. S., Rodrigues V. P., Benatti B. B. (2016). Possible evidence of systemic lupus erythematosus and periodontal disease association mediated by Toll-like receptors 2 and 4. *Clinical and Experimental Immunology*.

[110] Weinstein R. S., Manolagas S. C. (2000). Apoptosis and osteoporosis. *The American Journal of Medicine*.

[111] Xie X., Liu M., Meng Q. (2019). Angelica polysaccharide promotes proliferation and osteoblast differentiation of mesenchymal stem cells by regulation of long non-coding RNA H19: an animal study. *Bone & Joint Research*.

[112] Hong G., Zhou L., Shi X. (2017). Bajijiasu abrogates osteoclast differentiation via the suppression of RANKL signaling pathways through NF-*κ*B and NFAT. *International Journal of Molecular Sciences*.

[113] Meng F. H., Li Y. B., Xiong Z. L., Jiang Z. M., Li F. M. (2005). Osteoblastic proliferative activity of Epimedium brevicornum Maxim. *Phytomedicine: international journal of phytotherapy and phytopharmacology*.

